# The essential roles of *Mps1* in spermatogenesis and fertility in mice

**DOI:** 10.1038/s41419-021-03815-4

**Published:** 2021-05-24

**Authors:** Qiang Fang, Xue-Lin Chen, Lei Zhang, Ya-Bin Li, Tian-Zeng Sun, Chen-Xin Yang, Jian-Feng Chang, Xiao-Mei Yang, Feng Sun

**Affiliations:** Research Center for Translational Medicine, Shanghai East Hospital, School of Medicine, School of Life Sciences and Technology, Tongji University, Shanghai, 200092 China

**Keywords:** Cell division, Spermatogenesis

## Abstract

Monopolar spindle 1 (MPS1), which plays a critical role in somatic mitosis, has also been revealed to be essential for meiosis I in oocytes. Spermatogenesis is an important process involving successive mitosis and meiosis, but the function of MPS1 in spermatogenesis remains unclear. Here, we generated *Mps1* conditional knockout mice and found that *Ddx4*-cre-driven loss of *Mps1* in male mice resulted in depletion of undifferentiated spermatogonial cells and subsequently of differentiated spermatogonia and spermatocytes. In addition, *Stra8*-cre-driven ablation of *Mps1* in male mice led to germ cell loss and fertility reduction. Spermatocytes lacking *Mps1* have blocked at the zygotene-to-pachytene transition in the prophase of meiosis I, which may be due to decreased H2B ubiquitination level mediated by MDM2. And the expression of many meiotic genes was decreased, while that of apoptotic genes was increased. Moreover, we also detected increased apoptosis in spermatocytes with *Mps1* knockout, which may have been the reason why germ cells were lost. Taken together, our findings indicate that MPS1 is required for mitosis of gonocytes and spermatogonia, differentiation of undifferentiated spermatogonia, and progression of meiosis I in spermatocytes.

## Introduction

Spermatogenesis occurs within the testis seminiferous epithelium and consists of the following stages in mice: mitosis of spermatogonia, meiosis of spermatocytes, and maturation of haploid spermatozoa^1^. Initially, spermatogonial stem cells undergo a series of mitosis events and then differentiate into B-type spermatogonia. They then enter meiosis I, becoming primary spermatocytes, as indicated by STRA8 expression^2–4^. Meiosis I involves the stages leptotene, zygotene, pachytene, diplotene, and diakinesis, which can be distinguished by different expression patterns of SYCP3^5,6^. Moreover, at the leptotene stage, double-strand breaks occur and activate γH2AX and SPO11^7,8^, while at zygonema, RAD51 is highly enriched at the break sites in the chromosome strands^9^. During the pachytene stage, meiotic homologous chromosomes combine and form the synaptonemal complex. SYCP3 localizes along the lateral chromosome axis, and SYCP1 loads onto the center to connect the two homologous chromosomes^5,6,9^. Subsequently, diplonema occurs, which features sex bodies marked by γH2AX^10^, and is followed by diakinesis.

Monopolar spindle 1 (MPS1) is a kinase that is conserved from yeast to mammals that plays a critical role in preventing erroneous chromosome segregation through the spindle assembly checkpoint (SAC) complex^11–13^. During mitosis, MPS1 directly binds to unattached kinetochores through CDC80, after which it phosphorylates and recruits other SAC proteins, such as BUB3, BUBR1, and MAD2, to the kinetochore^14–17^. This process inhibits anaphase-promoting complex/cyclosome (APC/C) activation until all microtubules stably attach to the kinetochores at anaphase onset^18^. MPS1 has important functions not only in mitosis but also in meiosis^19^. In previous research, a mutant mouse strain was generated that expresses a form of ΔNMPS1 with a 107-amino acid deletion at the N-terminus. The N-terminus is involved in binding to the kinetochore^20,21^. Without MPS1 localization, precocious APC/C activation leads to the missegregation or nondisjunction events observed in mouse oocytes due to failure of SAC control and chromosome alignment in meiosis I^20^. Furthermore, MPS1 kinase activation is essential for cohesin protection in a manner dependent on the localization of SGO2 to the centromere in mouse oocytes^22^. However, whether *Mps1* plays an essential role in mouse spermatogenesis, especially in mitosis and male meiosis, is still unknown.

To investigate the function of MPS1 in the regulation of spermatogenesis, we generated two conditional *Mps1* knockout strains and found that *Mps1* is required for mitosis of gonocytes and spermatogonia, differentiation of undifferentiated spermatogonia, and progression of meiosis I in spermatocytes.

## Results

### *Mps1* was required for postnatal mitotic divisions of male germ cells

**Fig. 1:**
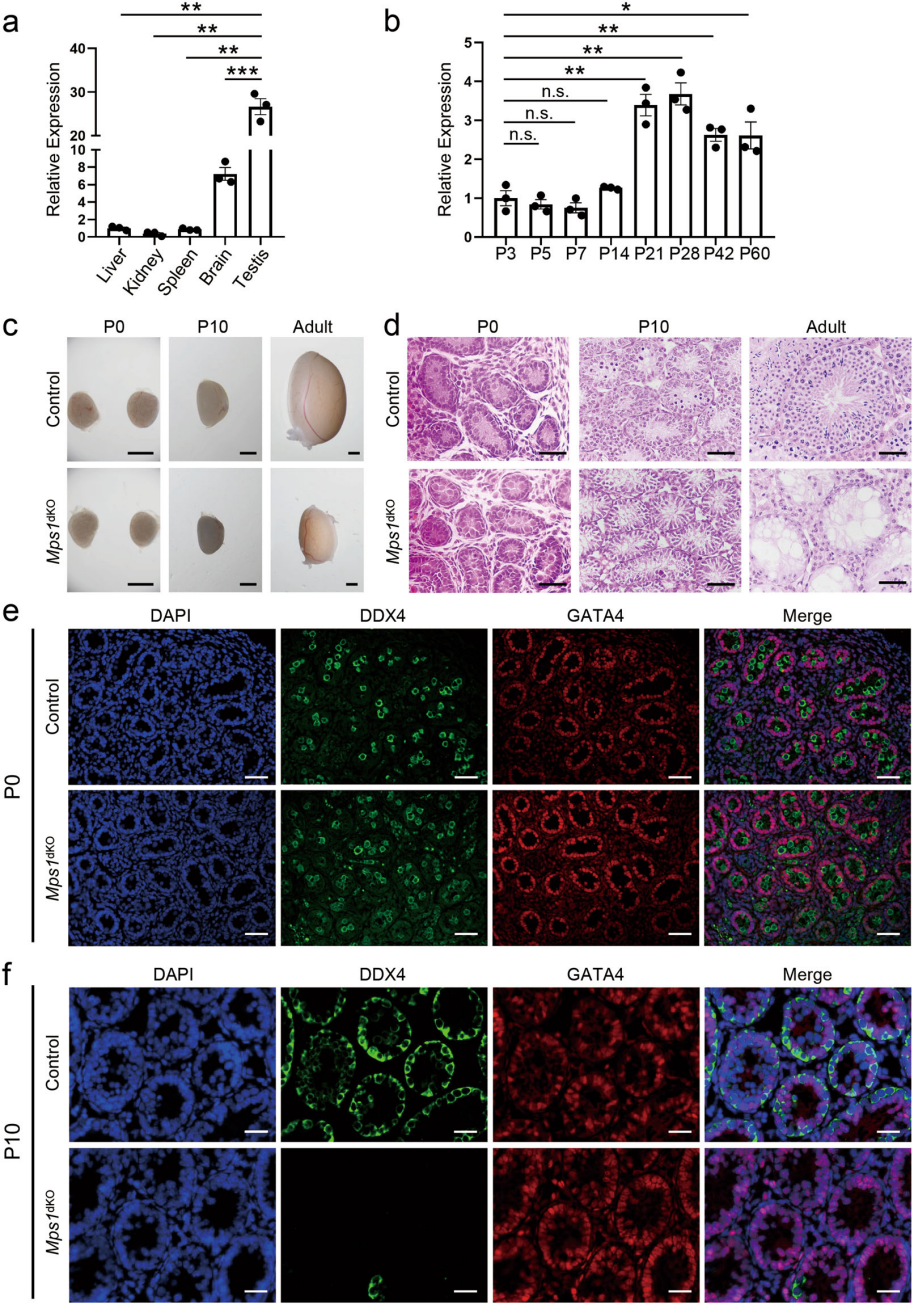
Ablation of *Mps1* in gonocytes and spermatogonia led to the depletion of germ cells. **a** qPCR analysis of *Mps1* expression level of liver, kidney, spleen, brain, and testis of wild-type mice at 2 month of age. *n* = 3; Student’s *t* test; **P < 0.01, ***P < 0.001. **b** qPCR analysis of *Mps1* expression level of wildtype mouse testes from P3 to P60. *n* = 3; Student’s *t* test; n.s. no significance, *P < 0.05, **P < 0.01. **c** Images of control and *Mps1*^dKO^ testes at P0, at P10, and during adulthood. Scale bar = 1 mm. **d** H&E staining of control and *Mps1*^dKO^ testes at P0, at P10, and during adulthood. Scale bar = 50 µm. **e** Immunofluorescence staining of DDX4 and GATA4 in control and *Mps1*^dKO^ testes at P0. Scale bar = 50 µm. **f** Immunofluorescence staining of DDX4 and GATA4 in control and *Mps1*^dKO^ testes at P10. Scale bar = 50 µm.

To explore *Mps1*’s function in spermatogenesis, we first characterized the spatiotemporal expression pattern of *Mps1* in mice. Quantitative polymerase chain reaction (qPCR) analysis of different adult tissues showed higher *Mps1* expression levels in testes than in other tissues (Fig. 1a). In testes, *Mps1* expression levels in the mouse testes aged from P3 (postnatal day 3) to P60 showed a dramatic increase from P21, when the male mice started to sexually mature (Fig. 1b). Using the published testes RNA-seq dataset from Soumillon et al.^23^, we further found that *Mps1* is especially enriched in spermatocytes (Fig. S1a). All these data suggested that *Mps1* may have important roles in spermatogenesis.

For studying the role of *Mps1*, we generated a conditional *Mps1-*knockout mouse strain. Specifically, we first generated *Mps1*^fl/fl^ mice by inserting two *LoxP* sites in intron 5 and intron 8 of the *Mps1* gene, which caused deletion of exons 6–8 and a frameshift mutation driven by Cre recombinase (Fig. S1b). Then, by mating *Mps1* floxed mice with *Ddx4*-Cre mice, which specifically express Cre recombinase in germ cells as early as embryonic day 15.5 (E15.5)^24^, we obtained germ cell-specific *Mps1*-knockout (*Mps1*^dKO^) mice. Successful knockout was verified by PCR (Fig. S1c, d).

The *Mps1*^dKO^ mice grew normally like the control mice, and we focused on the spermatogenesis of male mice. First, we examined the morphology of testes from newborn (P0) to adult mice (Fig. 1c). There was no difference between *Mps1*^dKO^ and control mice in terms of testis size at P0. At P10, the testes of *Mps1*^dKO^ and control mice started to become different in size, with the testes of *Mps1*^dKO^ mice being slightly smaller than those of control mice. The difference became much more significant in adult mice. Then, to determine the reason, we assessed the composition of cell types in seminiferous tubules, which are the sites of spermatogenesis (where germ cells develop into spermatozoa). Using hematoxylin–eosin (H&E) staining, we were able to distinguish two different types of cells on the basis of morphology, i.e., triangle-like Sertoli cells and round germ cells. At P0, both types of cells were visible in *Mps1*^dKO^ and control seminiferous tubules (Fig. 1d). However, there were few germ cells (round-shaped) left in the seminiferous tubules at P10, and no germ cells were present in adult *Mps1*^dKO^ mice; only triangle-like Sertoli cells remained (Fig. 1d). Moreover, many empty seminiferous tubules existed in adult *Mps1*^dKO^ mice. Consistently, no mature sperm were found in the cauda epididymides of *Mps1*^dKO^ mice (Fig. S1e).

To further identify the cell types that disappeared, we employed immunostaining on seminiferous tubule sections of P0 and P10 mice. We used two kinds of marker genes to distinguish cell types: GATA4 for Sertoli cells and DDX4 for germ cells. Consistent with the H&E staining results, DDX4-positive germ cells and GATA4-positive Sertoli cells were present in both control and *Mps1*^dKO^ P0 mice (Fig. 1e). However, almost no DDX4-positive germ cells were found in the *Mps1*^dKO^ P10 testes (Fig. 1f). Nonetheless, Sertoli cells (GATA4-positive) remained unaffected in the *Mps1*^dKO^ testes, as expected. Taken together, the results indicate that *Mps1* knockout in early-stage male germ cells results in severe germ cell loss. Previous studies have revealed the important roles of MPS1 in mitosis in somatic cells. Given that germ cells are generated via mitosis of gonocytes and spermatogonia, the findings suggest that the male germ cell depletion in *Mps1*^dKO^ mice might be due to disruption of postnatal mitosis.

### Postnatal disruption of *Mps1* led to severe defects in spermatogenesis and significantly reduced male fertility

Since knocking out *Mps1* in *Mps1*^dKO^ mouse testes triggered such dramatic germ cell loss that almost no spermatocytes could be observed, the role of MPS1 in the meiosis of spermatocytes remained unclear. To investigate the function of MPS1 in meiosis, we knocked out the *Mps1* gene in germ cells by mating *Stra8*-cre mice, which specifically express Cre recombinase at P3 (early spermatogonia) and exhibit increased expression until P7 (preleptotene spermatocytes) in testes^25^. This strain (*Mps1*^sKO^) was verified by PCR (Fig. S2a). The knockout efficiency of *Mps1* was confirmed by using Western blotting, which revealed that the protein levels of MPS1 were significantly reduced in the testes of the *Mps1*^sKO^ mice (Fig. S2b).

**Fig. 2:**
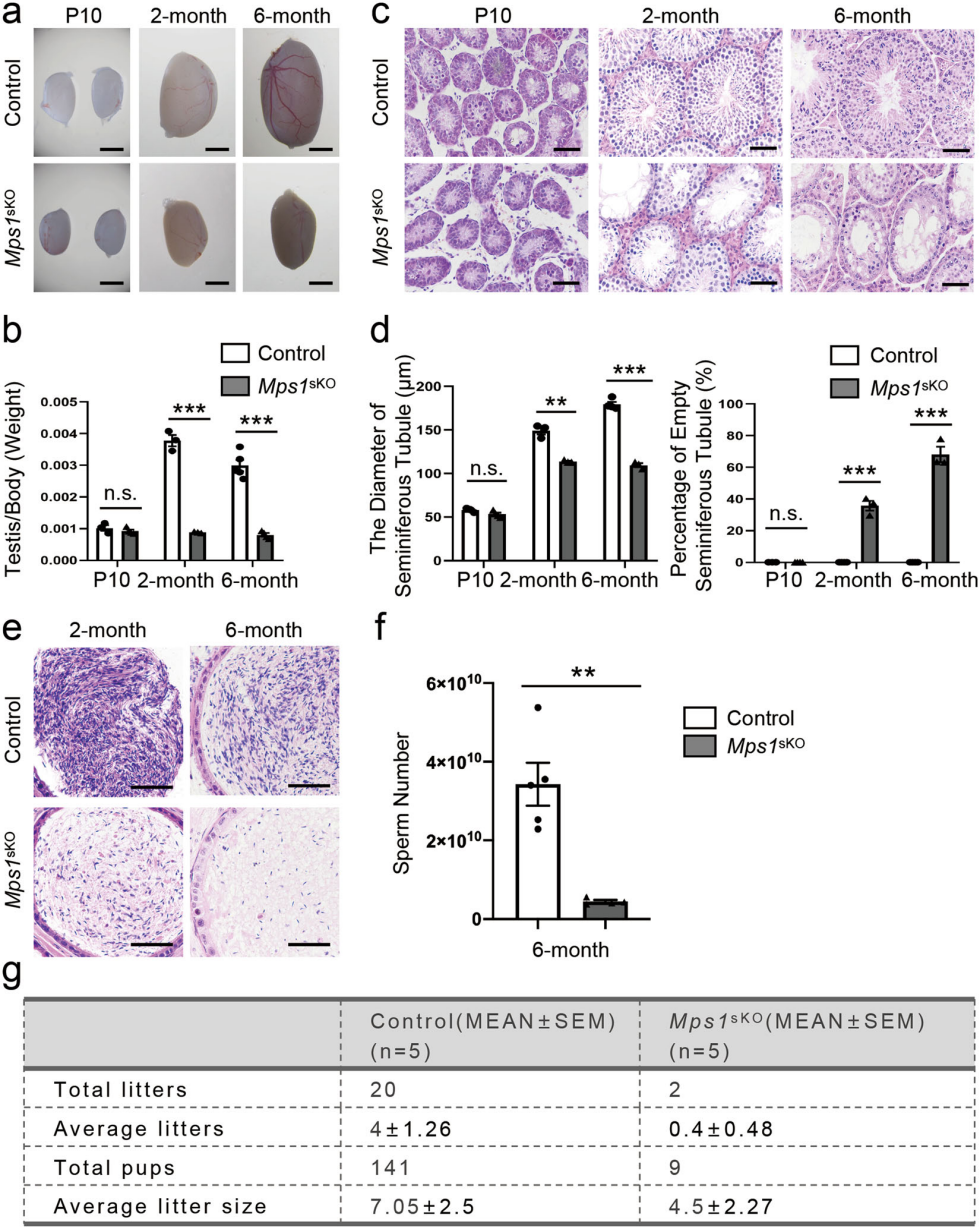
Postnatal disruption of *Mps1* led to severe defects in spermatogenesis and significantly reduced male fertility. **a** Image of control and *Mps1*^sKO^ testes at P10, 2 months of age, and 6 months of age. Scale bar = 2 mm. **b** Ratios of testis weight to body weight for control and *Mps1*^sKO^ mice at P10, 2 months of age, and 6 months of age. *n* ≥ 3; Student’s *t* test; n.s. no significance; ***P < 0.001. **c** H&E staining of control and *Mps1*^sKO^ testes at P10, 2 months of age and 6 months of age. Scale bar = 50 µm. **d** Statistical results for the seminiferous tubule diameters and empty seminiferous tubule percentages of control and *Mps1*^sKO^ testes at P10, 2 months of age, and 6 months of age. At least 100 tubules were counted from at least 3 different mice; Student’s *t* test; **P < 0.01, ***P < 0.001. **e** H&E staining of control and *Mps1*^sKO^ cauda epididymides from 2 and 6-month-old mice. Scale bar = 50 µm. **f** Sperm counts from the cauda epididymides of at least four different mice at 6-month of age. Student’s *t* test; **P < 0.01. **g** Fertility test of male control and *Mps1*^sKO^ mice. In each group, five 3-month-old male mice were individually mated with two wild-type female mice for 3 continuous months.

As we observed, the *Mps1*^sKO^ mice were mostly normal, but their testes were different from those of the control mice. The testes of *Mps1*^sKO^ and control mice were similar in size and mass at P10, but the testes of *Mps1*^sKO^ mice became smaller and lighter than those of control mice at 2 months and 6 months (Fig. 2a, b). Specifically, the testis weight/body weight ratio was significantly reduced from 0.37% in control mice to 0.12% in *Mps1*^sKO^ mice at 2 month (32.4%) and from 0.30% to 0.08% at 6 month (28.3%) (Fig. 2b). In addition, the epididymides were detectably smaller in *Mps1*^sKO^ mice than in control mice (Fig. S2c). Histologically, the seminiferous tubules of *Mps1*^sKO^ mice displayed decreased diameters, reduced cell numbers, and increased empty seminiferous tubule percentages at 2 and 6 months (Fig. 2c, d). The average diameter of *Mps1*^sKO^ seminiferous tubules was reduced to 76% and 61% of that of the control mice at 2 and 6 months, respectively (Fig. 2d). At 2 months and 6 months, 35% and 68% of *Mps1*^sKO^ seminiferous tubules were degenerated with aberrant spermatogenesis, respectively, while degenerated tubules were hardly detected in the control mice in the time window we examined (Fig. 2d).

Next, we examined whether *Stra8*-Cre-mediated *Mps1* ablation affected sperm production and male fertility. H&E staining of the cauda epididymis, which is the primary storage site for mature sperm, showed that the density of mature sperm was largely reduced in adult (2 months and 6 months) *Mps1*^sKO^ mice compared to control mice (Fig. 2e). To validate this finding, we released and counted all mature sperm from the cauda epididymides of 6-month-old mice. Strikingly, the average sperm number of *Mps1*^sKO^ mice was decreased to approximately one-tenth of that of control mice (Fig. 2f). We then assessed the fertility of male *Mps1*^sKO^ and control mice. Three-month-old male control and *Mps1*^sKO^ mice were bred with two adult wild-type females for 3 continuous months (*n* = 5). Whereas the control males produced litters of normal size over this period, the *Mps1*^sKO^ males showed significantly reduced fertility. The total number of litters produced by the control mice was 20 (average number of litters: 4 ± 1.26), and the total number of pups was 141 (average litter size: 7.05 ± 2.5). However, 3 of 5 *Mps1*^sKO^ mice were infertile during this time period, and another two *Mps1*^sKO^ mice each produced only one litter (average number of litters: 0.4 ± 0.49); the total pup number dropped to 9 (average litter size: 4.5 ± 2.27) (Fig. 2g). Thus, postnatal deletion of *Mps1* led to lower productivity of mature sperm and significantly reduced male fertility, suggesting that the reductions in average litter number and size might have been caused by reductions in sperm numbers in the testes of *Mps1*^sKO^ males.

### Deletion of *Mps1* within differentiating spermatogonia via *Stra8*-Cre revealed a role for *Mps1* in spermatogonial differentiation

**Fig. 3:**
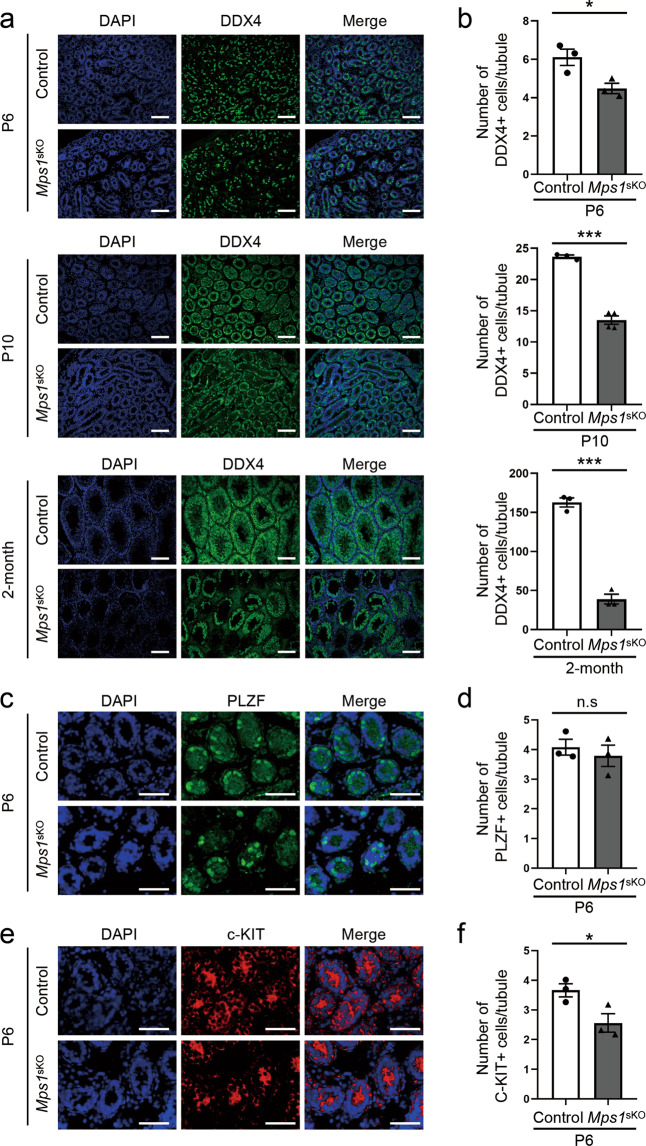
Deletion of *Mps1* reduced spermatogonial differentiation during spermatogenesis. **a** Immunofluorescence staining of DDX4 in testes of control and *Mps1*^sKO^ mice at P6, P10, and 2 months of age. Scale bar = 50 µm. **b** Statistical results for the number of DDX4-positive cells per tubule at P6, P10, and 2 months of age. *n* ≥ 3; Student’s *t* test; *P < 0.05, ***P < 0.001. **c** Immunofluorescence staining of PLZF in control and *Mps1*^sKO^ testes at P6. Scale bar = 50 µm. **d** Statistical results for the number of PLZF-positive cells per tubule at P6. *n* ≥ 3; Student’s *t* test; n.s. no significance. **e** Immunofluorescence staining of c-KIT in control and *Mps1*^sKO^ testes at P6. Scale bar = 50 µm. **f** Statistical results for the number of c-KIT-positive cells per tubule at P6. *n* ≥ 3; Student’s *t* test; *P < 0.05.

To determine the mechanism of severe cell loss and fertility reduction in *Mps1*^sKO^ mice, it was necessary to investigate which cell types were lost and when they began to disappear during spermatogenesis. We examined the expression of germ cell markers in testes of mice at P6, P10, and 2 months by immunostaining analysis. The DDX4 staining data showed that the number of *Mps1*^sKO^ mouse germ cells per seminiferous tubule had decreased to 66% and 57% of that in control mice by P6 and P10, respectively (Fig. 3a, b), although the testis weight did not differ, as previously mentioned (Fig. 2b). The impact was greater in 2-month-old *Mps1*^sKO^ mice, which retained only 24% of the germ cells in control mice. On the other hand, the number of PLZF-positive cells per tubule, representing undifferentiated spermatogonia, was the same between *Mps1*^sKO^ mice and controls at different ages, as expected (Fig. 3c, d; Fig. S3a, b). We further investigated the fates of *Mps1*^sKO^ spermatogonia via immunostaining for c-KIT, a marker of differentiated spermatogonia. The results indicated that the c-KIT-positive cell number in *Mps1*^sKO^ testes was reduced to 70% of that in control testes at P6, which suggested that spermatogonial differentiation may have been affected in *Mps1*^sKO^ mice (Fig. 3e, f). In summary, postnatal deletion of *Mps1* via *Stra8*-Cre does not affect the number of undifferentiated spermatogonia but plays a role in spermatogonial differentiation during spermatogenesis.

### Loss of *Mps1* compromised the progression of meiosis I and delayed the transition from zygotene to pachytene

**Fig. 4:**
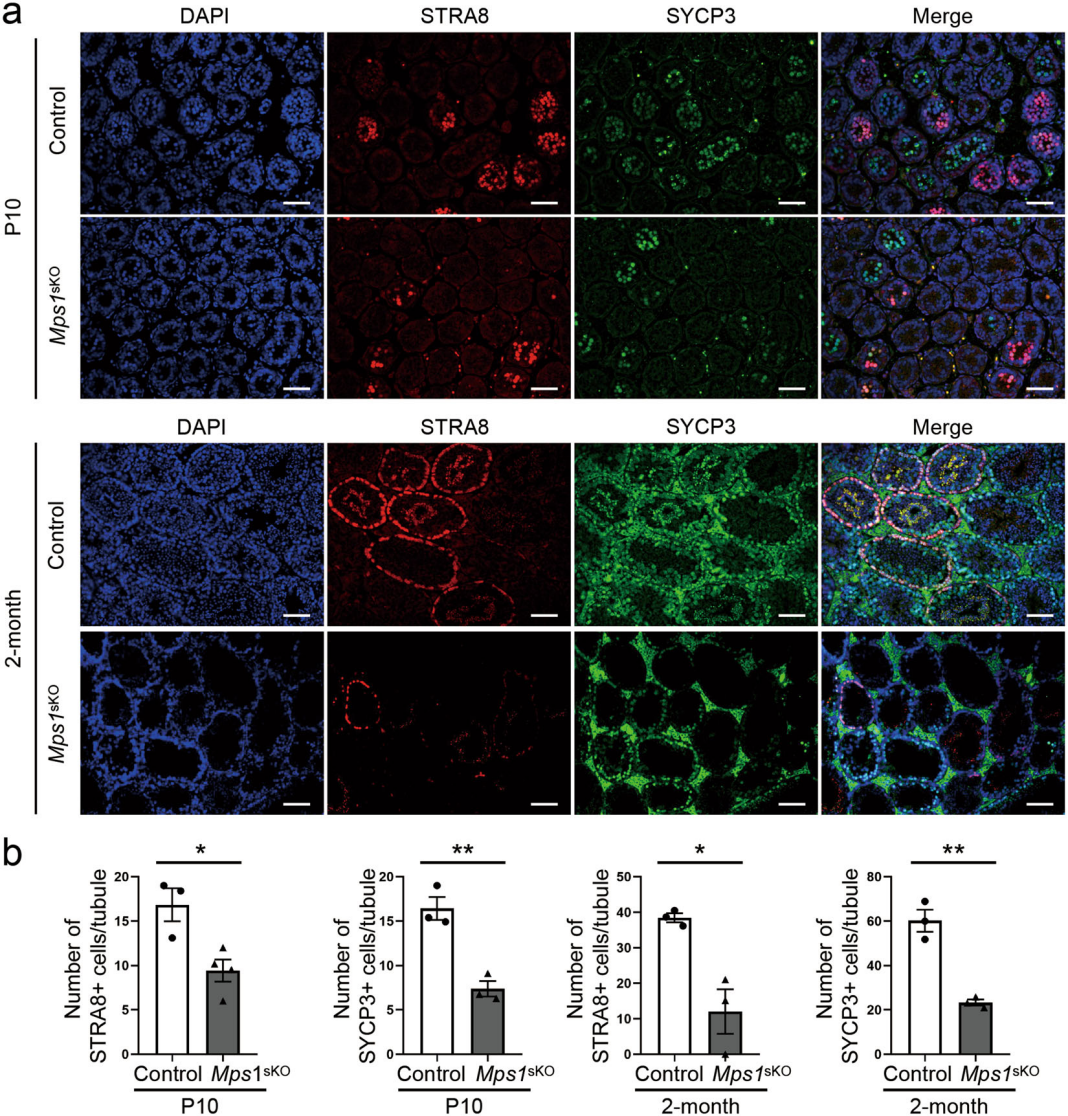
Loss of *Mps1* caused meiosis defects during spermatogenesis. **a** Immunofluorescence staining of STRA8 and SYCP3 in control and *Mps1*^sKO^ testes at P10 and 2 months of age. Scale bar = 50 µm. **b** Statistical results for the number of STRA8-positive and SYCP3-positive cells per tubule in testes from control and *Mps1*^sKO^ mice at P10 and 2 months of age. *n* ≥ 3; *P < 0.05, **P < 0.01.

Meiosis is a key feature of spermatogenesis that begins at approximately P10. STRA8 and SYCP3 are a marker of meiosis initiation and a synaptonemal complex component, respectively. Immunostaining analysis showed that the numbers of STRA8-expressing and SYCP3-expressing cells per seminiferous tubule in *Mps1*^sKO^ mice were significantly reduced to approximately 56% and 45% of those in control mice at P10. Similarly, the staining results at 2 month revealed that *Mps1*^sKO^ mice had only 30% of the STRA8-positive and 38% of the SYCP3-positive cells of control mice (Fig. 4a, b).

**Fig. 5:**
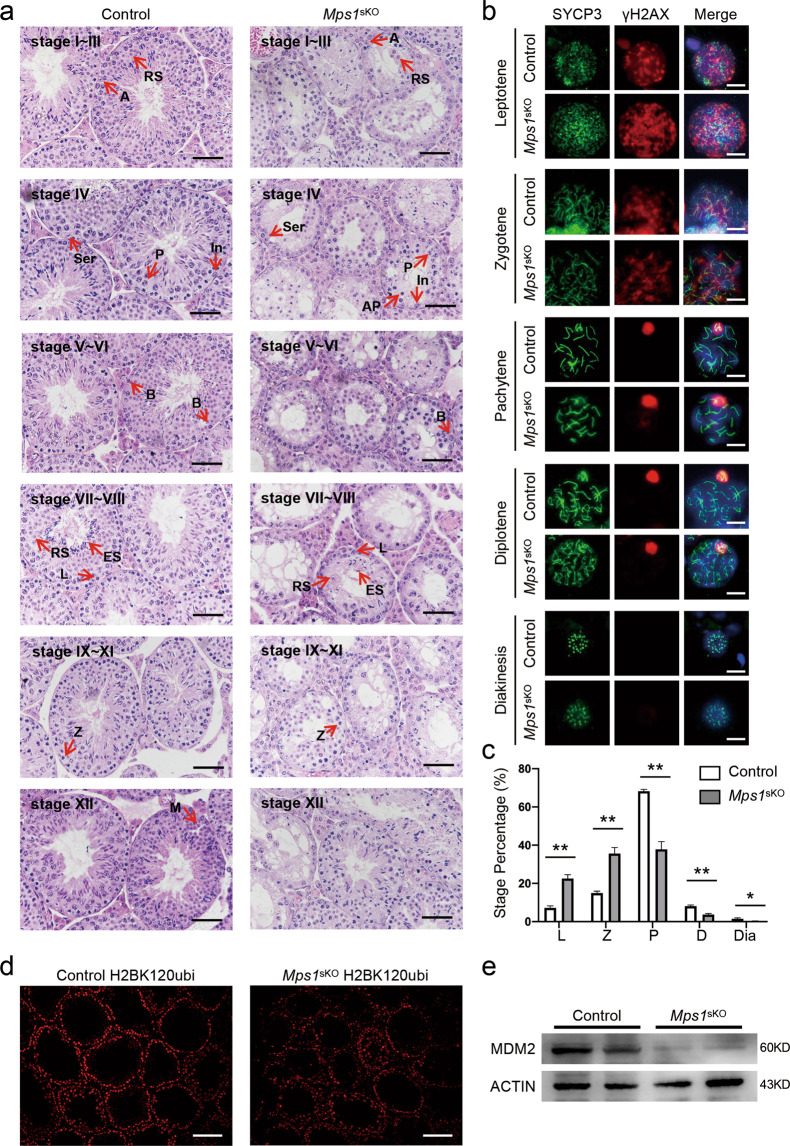
In *Mps1*^sKO^ mouse testes, the zygotene-to-pachytene transition was delayed, and the progression of meiosis I was compromised. **a** Representative H&E staining of control and *Mps1*^sKO^ seminiferous tubules. A type A spermatogonia, In intermediate spermatogonia, B type B spermatogonia, P pachytene spermatocytes, L leptotene spermatocytes, Z zygotene spermatocytes, RS round spermatids, ES elongated spermatids, M meiotic divisions, AP apoptosis pachytene, Ser Sertoli. Scale bar = 50 µm. **b** Meiotic spermatocyte spreads for control and *Mps1*^sKO^ mice at 1-month of age. Co-staining was performed with SYCP3 and γH2AX antibodies. Scale bar = 10 µm. **c** Percentage of meiotic spermatocytes in each stage. L leptotene, Z zygotene, P pachytene, D diplotene, Dia diakinesis. A total of 650–1000 spermatocytes were counted from 3 different mice. Student’s *t* test; *P < 0.05, **P < 0.01. **d** Immunofluorescence staining of H2BK120ubi in control and *Mps1*^sKO^ testes at 2 months of age. Scale bar = 100 µm. **e** Western blot of MDM2 in control and *Mps1*^sKO^ testes at 2 months of age.

According to the spermatocyte composition in the seminiferous tubules, spermatid development in mice has been divided into 12 stages^26,27^. To determine the exact stage of meiosis that was affected by *Mps1* knockout, we performed H&E staining on testes of 6-month-old mice to trace the seminiferous epithelium stages of the testicular tubules. We found that spermatogenesis seemed delayed at stage IV, during which pachytene spermatocytes should appear and localize inside of the seminiferous lumen. There were fewer pachytene spermatocytes (marked as P) in *Mps1*-deletion testes than in control testes. Apoptotic pachytenes (marked as AP) were also observed (Fig. 5a). Although some spermatocytes survived in stage IV, fewer round and elongated spermatids formed in *Mps1*^sKO^ testes than in control testes. In addition, meiotic spermatocytes (marked as M) were rarely observed in stage XII *Mps1*^sKO^ testes.

To further identify the delayed stages in the prophase of meiosis, meiotic stage progression was assessed from chromosomal spreads of spermatocytes that were prepared from testes of 1-month male mice and immunostained with anti-SYCP3 and anti-γH2AX antibodies (Fig. 5b, c). In both the *Mps1*^sKO^ and control groups, all five stages from leptotene to diakinesis were observed based on the immunostaining patterns of SYCP3 and γH2AX in spermatocytes. The proportion of pachytene spermatocytes was reduced from 68.25% in control mice to 37.85% in *Mps1*^sKO^ mice, while the proportions of spermatocytes in diplotene and diakinesis were reduced from 8.08% in control mice to 3.70% in *Mps1*^sKO^ mice and from 1.56% in control mice to 0.28% in *Mps1*^sKO^ mice, respectively. On the other hand, spermatocytes of *Mps1*^sKO^ mice accumulated in leptotene (control mice: 7.24%; *Mps1*^sKO^ mice: 22.55%) and in zygotene (control mice: 14.97%; *Mps1*^sKO^ mice: 35.61%). These data indicated that the transition of zygotene spermatocytes to pachytene spermatocytes was impaired. This finding was investigated further via examination of H3 core histone phosphorylation at serine 10 (H3pSer10). This type of phosphorylation is coincident with metaphase progression, and the signal then becomes more intense throughout chromatin during the diakinesis phase and the first meiotic metaphase (MI) in spermatocytes^28^. We found that the numbers of H3pSer10-positive germ cells were dramatically reduced in *Mps1*^sKO^ testes (Fig. S4a, b) and that the percentages of H3pSer10-positive metaphase and anaphase spermatocytes were significantly lower in tubule sections from *Mps1*^sKO^ mice than in those from control mice (Fig. S4b, right). This finding suggested that loss of *Mps1* compromised the progression of meiosis I.

Collectively, the evidence indicated that deletion of *Mps1* during meiosis resulted in a dramatic decrease in the number of germ cells, which may have been caused by defects in the meiosis process. In particular, the transition from the zygotene to the pachytene stage might have been delayed or partially disrupted in the spermatocytes of *Mps1*^sKO^ mice.

Previously, Xu et al.^6^ found H2B ubiquitination regulates the progression of meiosis I by promoting chromatin relaxation, and its E3 ligase *Rnf20* knockout mice had similar phenotypes, such as the smaller size of testes, loss of germ cells and spermatocytes arrested at the prophase of meiosis I. We examined the levels of H2BK120 mono-ubiquitination in the testes of control and *Mps1*^sKO^ mice and noticed that it decreased in *Mps1*^sKO^ testes at 2 months of age compared with that in control testes (Figs. 5d and S4c). MPS1 has been reported to regulate the level of H2BK120ubi mediated by MDM2, another E3 ubiquitin ligase promoting the ubiquitination of H2B^28,29^, and knockdown of *Mps1* by RNAi diminished MDM2 expression in U2OS cells^30^. As expected, the expression level of MDM2 was downregulated in *Mps1*^sKO^ testes, compared to that in control testes (Fig. 5e). In a word, loss of *Mps1* compromised the prophase of meiosis I progress which may be due to decreased H2B ubiquitination level mediated by MDM2.

### The expression of meiosis-related genes and apoptosis-related genes was altered in the testes of *Mps1*^sKO^ mice

**Fig. 6:**
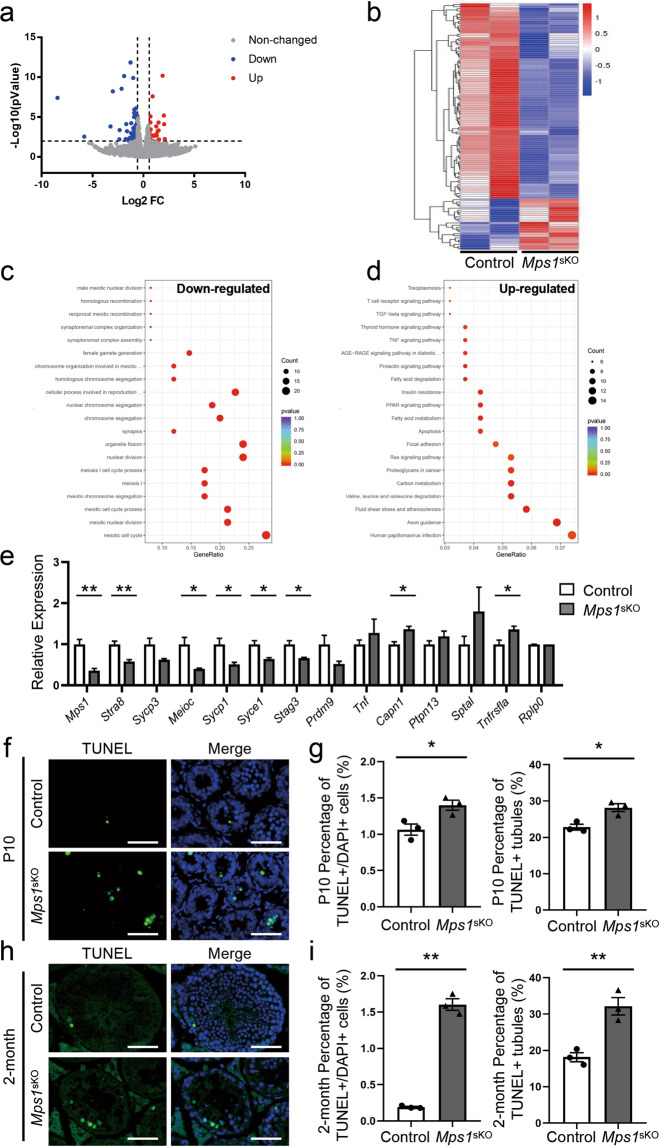
Loss of *Mps1* altered the expression of genes involved in meiosis and caused abnormal apoptosis in seminiferous tubules. **a** Gene expression pattern in *Mps1*^sKO^ testes compared with control testes at P10. An FC > 1.5 and a *P* value < 0.01 were used as the cut-offs. **b** Heatmap of the DEGs between control and *Mps1*^sKO^ testes at P10. In total, 92 genes were downregulated, and 24 genes were upregulated. An FC > 1.5 and a *P* value < 0.01 were used as the cut-offs. **c** Top 20 GO terms for the downregulated DEGs. An FC > 1.5 and a *P* value < 0.01 were used as the cut-offs. **d** Top 20 KEGG terms for the upregulated DEGs. An FC > 1.2 and a *P* value < 0.05 were used as the cut-offs. **e** qPCR analysis of DEGs involved in meiotic biological processes and apoptosis in *Mps1*^sKO^ testes at P10. Student’s *t* test; *n* = 3; *P < 0.05. **f** Immunofluorescence staining of TUNEL in control and *Mps1*^sKO^ testes at P10. **g** Statistical results for the percentage of TUNEL-positive cells among DAPI-positive cells per tubule and the percentage of TUNEL-positive seminiferous tubules in control and *Mps1*^sKO^ testes at P10. *n* ≥ 3; *P < 0.05. **h** Immunofluorescence staining of TUNEL in control and *Mps1*^sKO^ testes at 2 months of age. **i** Statistical results for the percentage of TUNEL-positive cells among DAPI-positive cells per tubule and the percentage of TUNEL-positive seminiferous tubules in control and *Mps1*^sKO^ testes at 2 months of age. *n* ≥ 3; **P < 0.01.

To investigated the changes in gene expression after *Mps1* deletion, we performed RNA sequencing of P10 testes from control and *Mps1*^sKO^ mice. We found that there were more downregulated genes than upregulated genes in *Mps1*^sKO^ mice (Fig. 6a and Table S2). Using cut-offs of a fold change greater than 1.5 and a *P* value less than 0.01, 92 genes were identified as downregulated, and 24 genes were identified as upregulated (Fig. 6b). We then performed Gene Ontology (GO) analysis to identify the enriched biological processes for each group of differentially expressed genes (DEGs). Notably, for the downregulated genes, the top ten enriched terms were all related to cell division. More importantly, seven of them were meiosis-related, including the meiotic cell cycle, synapsis, and chromosome segregation terms, among others. The most abundant term, the meiotic cell cycle, was enriched for 21 (22.8%) of the 92 downregulated genes (Fig. 6c and Table S3). To fully discover the biological pathways affected, we relaxed the cut-off for upregulated genes to 1.2-fold, and another 475 genes were identified as upregulated. Of these, 8 out of 189 genes were associated with the apoptosis term. Unsurprisingly, apoptosis stood out as one of the enriched Kyoto Encyclopedia of Genes and Genomes (KEGG) terms for the upregulated genes (Fig. 6d and Table S4). To validate the expression changes in these genes, we examined 13 of them (*Mps1*, *Stra8*, *Sycp3*, *Meioc*, *Sycp1*, *Syce1*, *Stag3*, *Prdm9*, *Tnf*, *Capn1*, *Ptpn13*, *Sptal*, and *Tnfrsfla*) by qPCR and detected consistent changes in *Mps1*^sKO^ testes (Fig. 6e). Therefore, the transcriptomic data indicated that widespread impacts on meiosis and apoptosis resulted from *Mps1* deletion.

### *Mps1* deletion elevated apoptosis in testes

In order to determine the fates of germ cells with *Mps1* loss, we conducted a TUNEL assay to investigate whether there were more cells undergoing apoptosis in *Mps1*^sKO^ testes than in control testes. On the one hand, there were more seminiferous tubules with TUNEL-positive cells in *Mps1*^sKO^ testes than in control testes (Fig. 6f–i). The percentage of TUNEL-positive tubules increased from 22.8% in control mice to 28.1% in *Mps1*^sKO^ mice at P10 and from 18.1% to 32.1% at 2 months. On the other hand, the percentage of TUNEL-positive cells per seminiferous tubule was also increased in the *Mps1*^sKO^ testes (Fig. 6f–i). At P10, on average, 1.06% of the cells in control seminiferous tubules were TUNEL positive, while 1.39% of the cells in *Mps1*^sKO^ tubules were TUNEL positive. At 2-month of age, the average percentage of TUNEL-positive cells was 0.19% in control tubules, while 1.60% in *Mps1*^sKO^ tubules. Overall, the TUNEL assay results showed that many more germ cells in seminiferous tubules underwent apoptosis in *Mps1*^sKO^ mice than in control mice, which indicated that the significant reductions in male germ cell numbers in *Mps1*^sKO^ mice were partly due to increased apoptosis.

## Discussion

MPS1, also known as TTK protein kinase, has been well studied in the context of mitosis in somatic cells^14,31^ and has also been reported to function in meiosis I during oogenesis^20^. Nevertheless, it remains unexplored whether *Mps1* plays roles in male spermatogenesis. Here, we explored MPS1 function in mouse spermatogenesis for the first time by utilizing conditional *Mps1* knockout in DDX4-positive and STRA8-positive germ cells and demonstrated that MPS1 is critical for spermatogonial differentiation and for the progression of meiosis I spermatocytes. We also revealed that *Mps1* may play its role partially by promoting male germ cell apoptosis.

STRA8 expression begins at P3 in the early stage of spermatogonia and lasts through the preleptotene stage^25^. *Stra8*-Cre mice have been used to investigate gene functions during male meiosis. In our study, we deleted *Mps1* in STRA8-positive male germ cells and revealed its function in the meiotic cell cycle. However, we also detected germ cell loss at P6 in *Mps1*^sKO^ seminiferous tubules. Germ cells at this stage included predominantly undifferentiated and differentiated spermatogonial cells that had not entered the meiotic process. Therefore, *Stra8*-Cre *Mps1* knockout affected not only meiosis but also the differentiation of spermatogonial cells. These results suggest that a spermatocyte-specific Cre knock-in mouse line may be more suitable than the knockout mouse line in the current study for research focusing exclusively on meiosis.

Apoptosis is a major form of programmed cell death critical for development and the damage response^32^. Activation of the TNF pathway can induce distinct cellular outcomes, including cell death, inflammation, and cell survival, in a context-dependent manner^33^. In our study, the TNF signaling pathway was upregulated in *Mps1*^sKO^ testes, which might have contributed to the increased apoptosis in *Mps1*^sKO^ testes. Given that loss of *Mps1* compromised the progression of meiosis I and increased apoptosis in seminiferous tubules, it seems likely that some germ cells that fail to complete meiosis I may undergo cell death in the absence of MPS1 expression.

Previous studies on *Mps1* function in cell division have mainly illustrated its function in the SAC. Hached and his colleagues revealed that *Mps1* functions in SAC control and chromosome segregation in oocyte meiosis I^20^. Our data demonstrate *Mps1*’s role in the prophase of the meiotic cell cycle, showing that *Mps1* ablation in this stage results in blockade of the zygotene-to-pachytene transition, which may be due to decreased H2B ubiquitination level mediated by MDM2. We also found that the percentages of H3pSer10-positive metaphase and anaphase spermatocytes were significantly decreased in tubule sections from *Mps1*^sKO^ mice, which suggests that *Mps1* may play a role in the progression from prophase to metaphase and anaphase. Further studies investigating the underlying mechanisms by which MPS1 plays roles in SAC control and chromosome segregation during male meiosis I are needed to complete our knowledge of MPS1 function in male germ cells.

## Materials and methods

### Animals

*Mps1* floxed conditional knockout mice were generated by targeted homologous recombination (Shanghai Model Organisms). Homology arms, *LoxP* sites, and selection markers were introduced into the targeting vector via a recombineering-based method. BAC DNA was purchased from the Sanger Institute. Then, the linearized vector was electroporated into ESCs and screened with G418 and Ganc. The correct clones were then injected into C57BL/6J blastocysts. After mating and identification by PCR with the primers listed below, we obtained *Mps1*-floxed mice. All mice and samples were selected randomly and proceeded in arbitrary order. No formal randomization techniques were used. All mice used and samples used in IF, H&E, sequencing analysis were only labeled with mouse ID numbers and no genotypes or treatments were indicated. Genotypes and treatments were labeled after the completion of data acquisition and analysis. The mice were handled according to the university guidelines, and all animal procedures were approved by the Tongji University Institutional Animal Care and Use Program Advisory Committee (protocol number: IACUC-017-006).

### Genotyping primers

The primers used to identify the *Mps1* floxed genotypes were *Mps1*-For (forward primer) and *Mps1*-Re (reverse primer). The floxed band was 353 bp, and the wild-type band was 167 bp. The primers used to identify the *Mps1* deletion alleles were *Mps1*-min-For and *Mps1*-Re, which produced a 319 bp band. The band for *Ddx4* was 240 bp, and that for *Stra8*-Cre was 180 bp. The primers for qPCR are listed in Table S1.

*Mps1*-For: TTGCTTGGTAGTTCTGTGGACT

*Mps1*-Re: AGCAGCTGTAAGTGCAGGAG

*Mps1*-min-For: CTGCGCTAACCTTGTGTTGG

*Stra8*-For: GTGCAAGCTGAACAACAGGA

*Stra8*-Re: AGGGACACAGCATTGGAGTC

*Ddx4-*For: CACGTGCAGCCGTTTAAGCCGCGT

*Ddx4*-Re: TTCCCATTCTAAACAACACCCTGAA.

### Histology and immunofluorescence

For paraffin sectioning, testes were fixed in Bouin’s trichrome at 4 °C overnight. After washing in phosphate-buffered saline (PBS), the testes were dehydrated with gradient concentrations of ethanol and xylene, embedded in paraffin, and cut into 5 μm-thick sections. H&E staining was performed and was followed by deparaffinization and rehydration. The slides were imaged with a Nikon microscope.

For frozen sectioning, testes were fixed in 4% PFA solution at 4 °C overnight and then dehydrated with 30% sucrose solution for another 2 days. The testes were dipped in O.C.T. compound and then quickly frozen in liquid nitrogen. Frozen sections with a thickness of 7 μm were subjected to heat-induced antigen retrieval (sodium citrate, pH = 6) in a water bath for 20 min at 98 °C. The sections were cooled down slowly in retrieval buffer until they reached room temperature. Then, they were blocked and permeated in 5% bovine serum albumin with 0.2% Triton in PBS for 1 h before being incubated with primary antibodies overnight at 4 °C. After washing in PBS three times, the sections were incubated with secondary antibodies at room temperature for 2 h. After staining with DAPI (Beyotime) for 5 min, the sections were mounted with a fluorescent mounting medium (Beyotime). All steps were performed with protection from light. Images were obtained with an Olympus microscope and processed with ImageJ and Photoshop software.

### Spermatocyte surface spreading

Testes from 1-month-old mice were subjected to a spermatocyte surface spreading assay as described by the previous research^34^. Briefly, the tunica albuginea was removed, and the tubules were placed into the hypotonic buffer (30 mM Tris, 50 mM sucrose, 17 mM trisodium citrate dihydrate, 5 mM EDTA, 0.5 mM DTT, and 0.5 mM PMSF, pH = 8.2) for 30–60 min. Subsequently, the tubules were torn into pieces in sucrose buffer (100 mM sucrose, pH = 8.2) and pipetted repeatedly to make a suspension. The cell suspension was smeared onto glass slides containing 1% PFA and 0.15% Triton X-100, pH = 9.2. The slides were dried overnight in a closed box with high humidity. Finally, the slides were washed twice for 2 min in 0.4% Photoflo (Kodak) and dried at room temperature. The slides were kept at −20 °C.

### Antibody

The antibody used in immunofluorescence staining were as follows: anti-DDX4 (ab13840, Abcam), anti-GATA4 (ab124265 Abcam), anti-c-KIT (25-1171-82, eBioscience) anti-SYCP3 (ab97672, Abcam), anti-STRA8 (ab49602, Abcam), anti-γH2AX (ab2893, Abcam), anti-H3pSer10 (ab5176, Abcam), anti-MPS1 (ab11108, Abcam), anti-H2BK120ubi (#5546, CST), anti-MDM2 (sc-965, Santa Cruz), anti H2B (ab1790, Abcam), and anti-ACTIN (sc-47778, Santa Cruz). All fluorophore-conjugated secondary antibodies were obtained from Jackson Immuno Research.

### RNA-Seq and analysis

Total RNA was extracted from whole testes at P10. RNA-Seq libraries were constructed from 1 μg of RNA using a NEBNext Ultra^TM^ RNA Library Prep Kit (NEB, USA) following the manufacturer’s instructions. Sequencing was performed on an Illumina NovaSeq platform with 150 bp paired-end reads. DEG analysis was performed with the DESeq2 R package. GO and KEGG enrichment analysis of the DEGs was implemented with the clusterProfiler R package. Libraries constructing, sequencing, and data analyzing were performed by Novogene Co., Ltd. (Tianjin, China).

### Sperm counting

Cauda epididymides from 6-month-old mice were removed and cut in 500 μl of DMEM. The medium was incubated at 37 °C for 7 min to release sperm. After diluting them 50 times, the sperm were analyzed with a cell counter (Countstar).

### Assessment of fertility

To test fertility, 3-month-old conditional knockout and control males were paired with two random adult wild-type females for at least three months. The numbers of offspring from each pregnancy were recorded.

### TUNEL assay

A TUNEL assay was performed following the instructions of an Apoptosis Detection Kit (Millipore). Sections after deparaffinization and rehydration were incubated with proteinase K (20 μg/ml) for 15 min and washed 2 times in PBS. Equilibration buffer was immediately applied and kept on the sections for at least 15 s. The excess liquid was gently tapped off, and the working-strength TDT enzyme was pipetted onto each section. The sections were incubated in a humidified chamber at 37 °C for 1 h and then placed in a stop buffer for 10 min. After washing 3 times in PBS, the sections were incubated with an anti-digoxigenin conjugate for 30 min at room temperature while protected from light and were washed in PBS 3 times again. After staining with DAPI (Beyotime) for 5 min, the sections were mounted with a fluorescent mounting medium. The slides were viewed with a Leica fluorescence microscope.

### Statistical analysis

All statistical data were analyzed by using GraphPad Prism. Sample sizes were described in figure legends. The variances of the two groups were compared by one-way ANOVA, and the *P* value was determined with a two-tailed unpaired Student’s *t* test or with Welch correction. Results were shown as mean ± s.e.m. Significance was shown as *P < 0.05, **P < 0.01, ***P < 0.001.

## Supplementary information

Supplemental Material

Table S1

Table S2

Table S3

Table S4

## Data Availability

All sequencing data that support the findings of this study have been deposited in the NCBI Gene Expression Omnibus (GEO) under accession number GSE165143.
