# Supplementary material for: The essential roles of Mps1 in spermatogenesis and fertility in mice
Source: Cell Death Dis. 2021 May 24;12(6):531. doi: 10.1038/s41419-021-03815-4 (PMC8144579; doi:10.1038/s41419-021-03815-4)
Supplement: Supplementary file 1 — Supplemental Material [file 41419_2021_3815_MOESM1_ESM.docx]

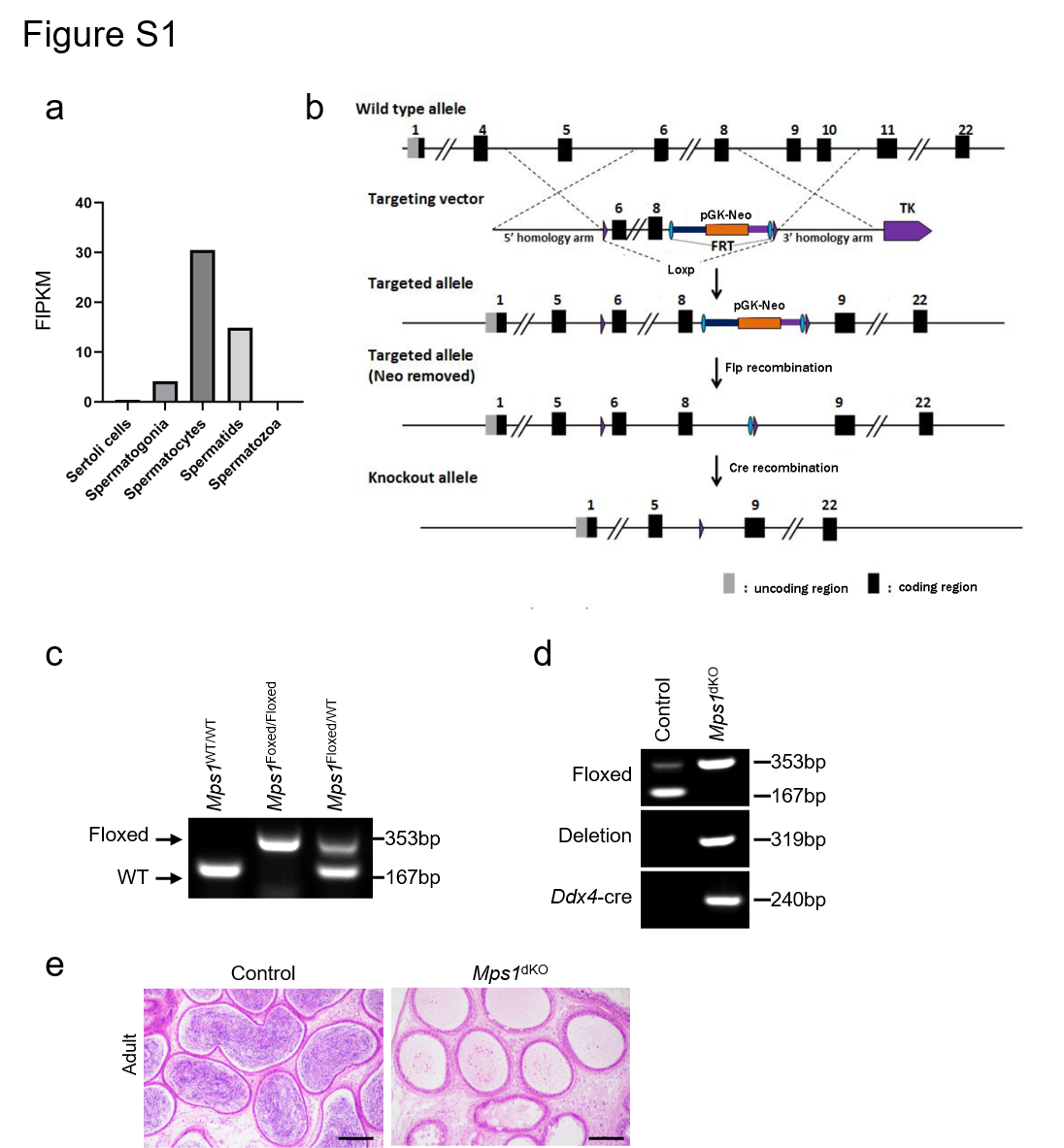


**Fig. S1 Generation of *Mps1* conditional knockout mice.**

(a) FPKM of *Mps1* expression in different cell types of testes^1^.

(b) Targeting strategy for generation of *Mps1* conditional knockout mice.

(c) Genotype of *Mps1* floxed mice. Floxed: 353 bp, wild-type: 167 bp.

(d) Genotype of *Mps1*^dKO^ mice. Floxed: 353 bp; wild-type: 167 bp; floxed after depletion: 319 bp; *Ddx4*-cre: 240 bp.

(e) H&E staining of adult control and *Mps1*^dKO^ cauda epididymides. Scale bar=50 µm.


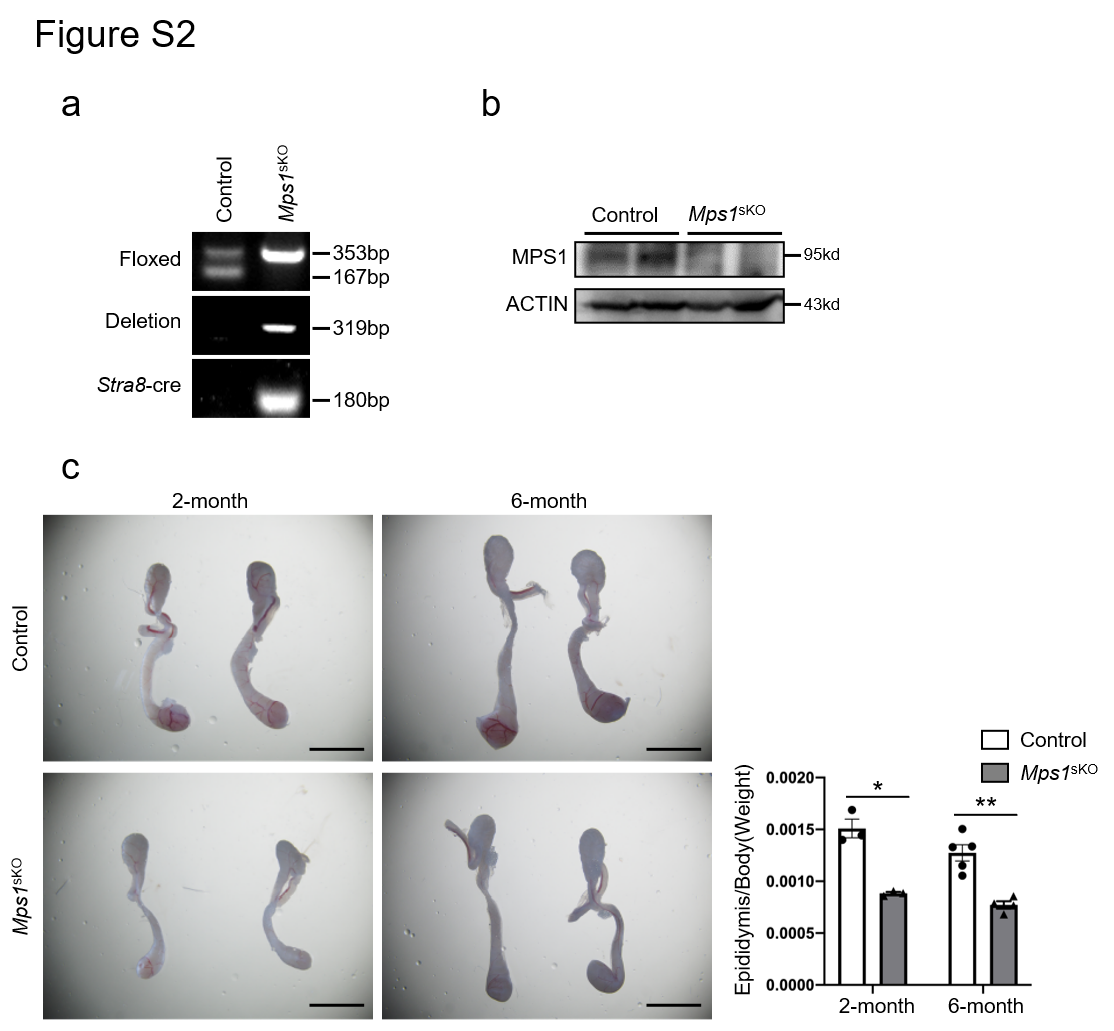


**Fig. S2 Generation and confirmation of *Mps1*^sKO^ mice**

(a) Genotypes of control and *Mps1*^sKO^ mice. Floxed: 353 bp; wild-type: 167 bp; Deletion: 319 bp after depletion; *Stra8*-cre: 180 bp.

(b) Western blot analysis of MPS1 expression levels in testes of 2-month-old control and *Mps1*^sKO^ mice.

(c) Images of epididymides from 2- and 6-month-old control and *Mps1*^sKO^ mice. And statistical results for the epididymis weight/body weight ratio in testes from 2-month-old and 6-month-old control and *Mps1*^sKO^ mice. n≥3; Student’s *t*-test; *P<0.05, **P<0.01.

Figure S3


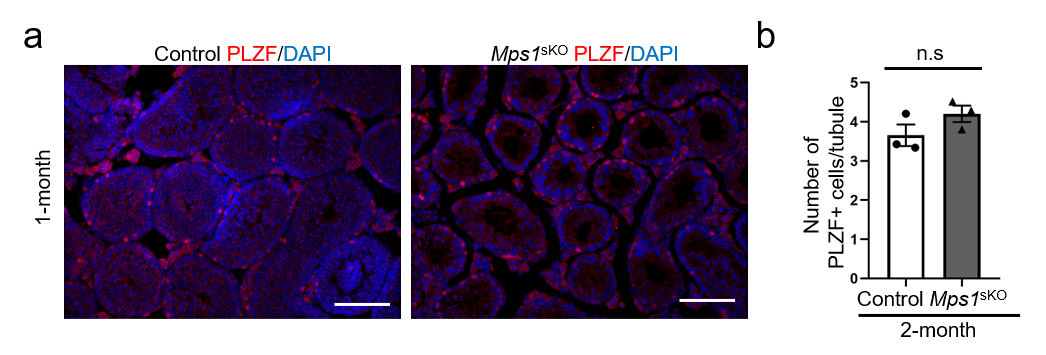


**Fig. S3 *Stra8*-driven *Mps1* deletion had no effect on the number of undifferentiated spermatogonia**

(a) Immunofluorescence staining of PLZF in testes from 1-month-old control and *Mps1*^sKO^ mice. Scale bar=100 µm.

(b) Statistical results for the number of PLZF-positive cells per tubule in testes from 1-month-old control and *Mps1*^sKO^ mice. n≥3; n.s., no significance.


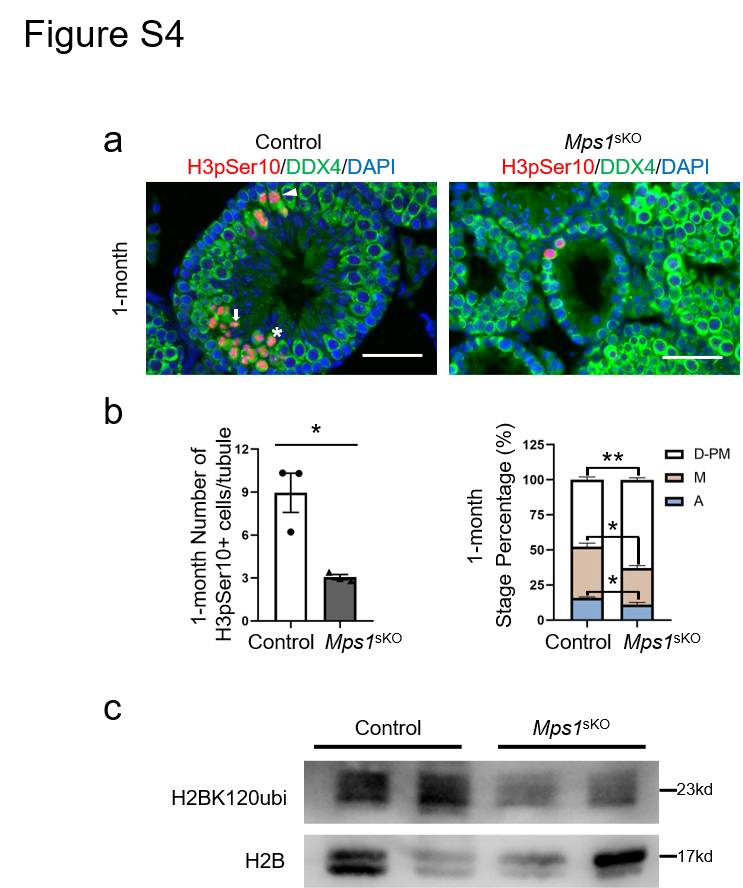


**Fig. S4 *Stra8*-driven *Mps1* deletion affected metaphase progression**

(a) Immunofluorescence co-staining of H3pSer10 and DDX4 in testes from 1-month-old control and *Mps1*^sKO^ mice. The arrowhead indicates the diplotene-to-pre-metaphase (D-PM) transition, the arrow indicates metaphase (M), and the asterisk indicates anaphase (A). Scale bar=50 µm.

(b) Statistical results for the number of H3pSer10-positive cells per tubule (left) and the percentage of H3pSer10-positive cells in each stage in testes from 1-month-old control and *Mps1*^sKO^ mice (right). n≥3; *P<0.05, **P<0.01.

(c) Western-blot of H2BK120ubi in testes of control and *Mps1*^sKO^ mice at 2-month-old of age.

**References**

1. Soumillon M, Necsulea A, Weier M, Brawand D, Zhang X, Gu H*, et al.* Cellular source and mechanisms of high transcriptome complexity in the mammalian testis. *Cell Rep* 2013, **3**(6)**:** 2179-2190.
